# Assessment of available anatomical characters for linking living mammals to fossil taxa in phylogenetic analyses

**DOI:** 10.1098/rsbl.2015.1003

**Published:** 2016-05

**Authors:** Thomas Guillerme, Natalie Cooper

**Affiliations:** 1 School of Natural Sciences, Trinity College Dublin, Dublin 2, Ireland; 2 Department of Life Sciences, Natural History Museum, Cromwell Road, London SW7 5BD, UK

**Keywords:** total evidence method, phylogenetic clustering, discrete morphological matrix, extinct, topology

## Abstract

Analyses of living and fossil taxa are crucial for understanding biodiversity through time. The total evidence method allows living and fossil taxa to be combined in phylogenies, using molecular data for living taxa and morphological data for living and fossil taxa. With this method, substantial overlap of coded anatomical characters among living and fossil taxa is vital for accurately inferring topology. However, although molecular data for living species are widely available, scientists generating morphological data mainly focus on fossils. Therefore, there are fewer coded anatomical characters in living taxa, even in well-studied groups such as mammals. We investigated the number of coded anatomical characters available in phylogenetic matrices for living mammals and how these were phylogenetically distributed across orders. Eleven of 28 mammalian orders have less than 25% species with available characters; this has implications for the accurate placement of fossils, although the issue is less pronounced at higher taxonomic levels. In most orders, species with available characters are randomly distributed across the phylogeny, which may reduce the impact of the problem. We suggest that increased morphological data collection efforts for living taxa are needed to produce accurate total evidence phylogenies.

## 1. Introduction

There is an increasing consensus among biologists that studying both living and fossil taxa is essential for fully understanding macroevolutionary patterns and processes [1,2]. To perform such analyses, it is necessary to combine living and fossil taxa in phylogenetic trees. One increasingly popular method, the total evidence method [3], combines molecular data from living taxa and morphological data from both living and fossil taxa in a supermatrix that can then be used with the tip-dating method [1,3–6], producing a chronogram with living and fossil taxa at the tips. A downside of this method is that it requires molecular data for living taxa and discrete morphological/anatomical data shared among both living and fossil taxa (i.e. hard tissue characters such as skeletal features). Sections of these data can be difficult, or impossible, to collect for every taxon in the analysis. For example, fossils rarely have molecular data and incomplete fossil preservation may reduce the number of anatomical characters available. Additionally, it has become less common to collect anatomical characters for living taxa when molecular data are available (e.g. in [7], only 13% of living taxa have coded anatomical characters). Unfortunately, these missing data can lead to errors in phylogenetic inference. We might expect the total evidence method to perform poorly when there is little overlap between coded anatomical characters in living and fossil taxa, because fossil taxa cannot be correctly placed within a clade of living species with no coded characters. Furthermore, simulations show that fossils are more likely to be placed in clades for which more characters have been coded, regardless of whether this is the correct clade [8].

The above-mentioned issues highlight that it is crucial to have sufficient coded anatomical characters available for living taxa in a clade before using the total evidence approach. However, it is unclear how many coded anatomical characters are actually available for living taxa, i.e. already coded from museum specimens and deposited in phylogenetic matrices accessible online, and how these data are distributed across clades. Intuitively, most people assume that these data have already been collected, but empirical analyses suggest otherwise (e.g. in [3,6,7]). To investigate this further, we assess the number of available coded anatomical characters for living mammals to determine whether enough data exist to build reliable total evidence phylogenies. We also determine whether the characters are phylogenetically overdispersed or clustered across mammalian orders.

## 2. Material and methods

### (a) Data collection and standardization

We downloaded all discrete morphological matrices containing any living and/or fossil mammal taxa from three major public databases: MorphoBank (morphobank.org [9]), Graeme Lloyd's website (graemetlloyd.com/matrmamm.html) and Ross Mounce's GitHub repository (github.com/rossmounce/cladistic-data). We also performed a systematic Google Scholar search for matrices that were not uploaded to these databases (see electronic supplementary material S1 for details). In total, we downloaded 286 matrices containing 5228 unique operational taxonomic units (OTUs). We used OTUs rather than species, because entries in the matrices ranged from species to families. We standardized the taxonomy as described in the electronic supplementary material, S1 and excluded OTUs that were not present in the phylogeny of [10] or the taxonomy of [11] to remove fossil species. This resulted in 1601 unique OTUs from 286 matrices.

## 3. Data availability and distribution

To assess the availability of coded anatomical characters for each mammalian order and across mammals, we calculated the percentage of OTUs with coded anatomical characters at three different taxonomic levels: family, genus and species. We do not distinguish between soft and hard characters, but the majority of matrices contain at least some hard tissue characters. We consider orders with less than 25% of living taxa with available anatomical characters as having low data coverage, and orders with more than 75% of living taxa with available anatomical characters as having high data coverage.

For each order and for all mammals, we investigated whether the available coded anatomical characters were (i) randomly distributed, (ii) overdispersed or (iii) clustered, with respect to phylogeny, using two metrics from community phylogenetics: the nearest taxon index (NTI; [12]) and the net relatedness index (NRI; [12]). NTI is most sensitive to clustering or overdispersion near the tips, whereas NRI is more sensitive to them across the whole phylogeny [13]. Both metrics were calculated using the picante package in R [14,15].

NTI is based on mean nearest neighbour distance (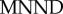) and is calculated as follows

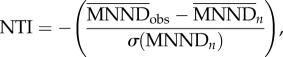
where 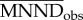 is the observed mean sum of the branch lengths between each of *n* taxa with available coded anatomical characters and its nearest neighbour with available coded anatomical characters in the phylogeny, 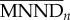 is the mean of 1000 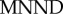 between *n* randomly drawn taxa, and 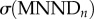 is the standard deviation of these 1000 random 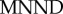 values. NRI is calculated in the same way, but using the mean phylogenetic distance (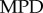):

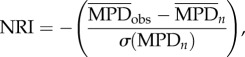
where 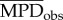 is the observed mean phylogenetic branch length of the tree containing only the *n* taxa with available coded anatomical characters. Negative NTI and NRI values show that the focal taxa are more overdispersed across the phylogeny than expected by chance, and positive values reflect clustering.

We calculated NTI and NRI values for all mammals or each mammalian order separately, at each different taxonomic-level. For each analysis, our focal taxa were those with available coded anatomical characters at that taxonomic-level and the phylogeny was the order pruned from [10].

## 4. Results

Across mammals, species coverage was low (less than 25% of species with available coded anatomical characters), but family coverage was high (more than 75% of families with available coded anatomical characters). For each order, 11 out of 28 had low coverage and seven had high coverage at the species-level. At the genus-level, one order had low coverage and 15 had high coverage, and at the family-level, no orders had low coverage and 25 had high coverage (table 1).

**Table 1.** Number of taxa with available discrete morphological data for mammalian orders at three taxonomic levels. The left vertical bar represents low coverage (<25%; dark grey (blue online)); the right vertical bar represents high coverage (>75%; light grey (orange online)). Negative net relatedness index (NRI) and nearest taxon index (NTI) values indicate phylogenetic overdispersion; positive values indicate phylogenetic clustering. Significant NRI or NTI values are in italics. *p < 0.05; **p < 0.01. (Online version in colour.)

order	taxonomic level	proportion of taxa	coverage	NRI	NTI
Mammalia (class)	family	129/148	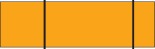	−1.19	1.09
*Mammalia (class)*	*genus*	*517/1186*	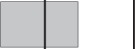	*−5.19*	3.71**
*Mammalia (class)*	*species*	*847/5017*	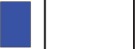	*−7.75*	3.54**
Afrosoricida	family	2/2	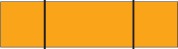		
Afrosoricida	genus	17/17	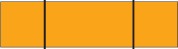		
Afrosoricida	species	23/42	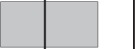	1.52	1.1
Carnivora	family	14/15	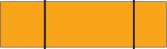	0.65	0.55
*Carnivora*	*genus*	*52/125*	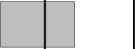	4.27**	*1.26*
*Carnivora*	*species*	*75/283*	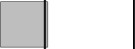	7.24**	*0.8*
Cetartiodactyla	family	21/21	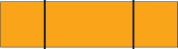		
Cetartiodactyla	genus	97/128	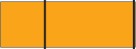	0.7	1.28
*Cetartiodactyla*	*species*	*169/310*	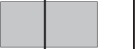	1.82*	*−0.24*
Chiroptera	family	15/18	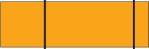	−0.23	0.61
*Chiroptera*	*genus*	*92/202*	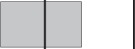	13.07**	*0.99*
*Chiroptera*	*species*	*214/1053*	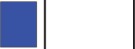	9.21**	*1.27*
Cingulata	family	1/1	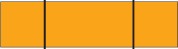		
Cingulata	genus	8/9	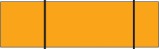	1.48	−1.54
*Cingulata*	*species*	*9/29*	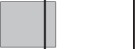	2.06*	*0.2*
Dasyuromorphia	family	2/2	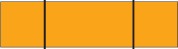		
Dasyuromorphia	genus	8/22	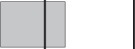	−0.78	−1.06
Dasyuromorphia	species	9/64	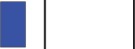	−0.86	−0.37
Dermoptera	family	1/1	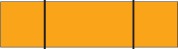		
Dermoptera	genus	1/2	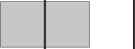		
Dermoptera	species	1/2	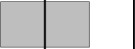		
Didelphimorphia	family	1/1	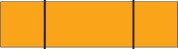		
Didelphimorphia	genus	16/16	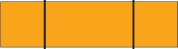		
Didelphimorphia	species	42/84	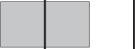	−1.61	0.12
Diprotodontia	family	11/11	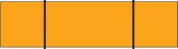		
Diprotodontia	genus	25/38	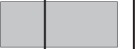	−1.15	−1.33
Diprotodontia	species	31/126	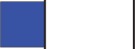	0.44	−1.79
Erinaceomorpha	family	1/1	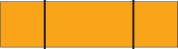		
Erinaceomorpha	genus	10/10	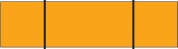		
Erinaceomorpha	species	21/22	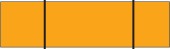	−1.04	−0.25
Hyracoidea	family	1/1	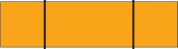		
Hyracoidea	genus	1/3	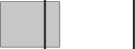		
Hyracoidea	species	1/4	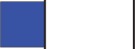		
Lagomorpha	family	2/2	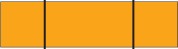		
Lagomorpha	genus	5/12	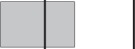	−0.95	−0.94
Lagomorpha	species	12/86	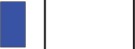	−0.62	−1.96
Macroscelidea	family	1/1	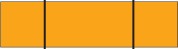		
Macroscelidea	genus	4/4	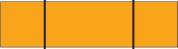		
Macroscelidea	species	12/15	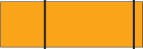	−1.24	−1.2
Microbiotheria	family	1/1	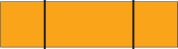		
Microbiotheria	genus	1/1	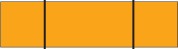		
Microbiotheria	species	1/1	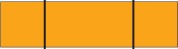		
Monotremata	family	2/2	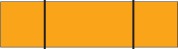		
Monotremata	genus	2/3	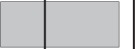	−0.68	−0.69
Monotremata	species	2/4	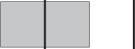	−1.01	−1
Notoryctemorphia	family	1/1	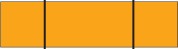		
Notoryctemorphia	genus	1/1	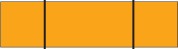		
Notoryctemorphia	species	0/2	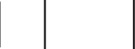		
Paucituberculata	family	1/1	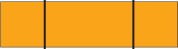		
Paucituberculata	genus	3/3	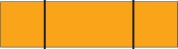		
Paucituberculata	species	5/5	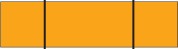		
Peramelemorphia	family	2/2	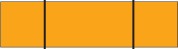		
Peramelemorphia	genus	7/7	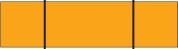		
Peramelemorphia	species	16/18	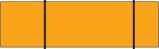	−0.14	0.91
Perissodactyla	family	3/3	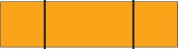		
Perissodactyla	genus	6/6	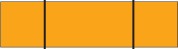		
Perissodactyla	species	10/16	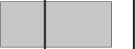	−0.1	−2.77
Pholidota	family	1/1	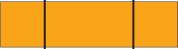		
Pholidota	genus	1/1	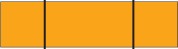		
Pholidota	species	4/8	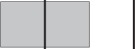	1.14	0.97
Pilosa	family	4/5	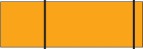	2.01	1.96
Pilosa	genus	4/5	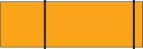	−0.91	0.36
*Pilosa*	*species*	*5/29*	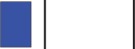	*1.18*	2.35**
Primates	family	15/15	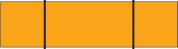		
Primates	genus	48/68	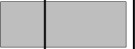	−0.37	−1.39
Primates	species	64/351	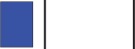	−0.66	−1.4
Proboscidea	family	1/1	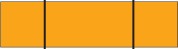		
Proboscidea	genus	2/2	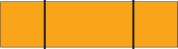		
Proboscidea	species	2/3	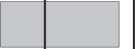	−0.67	−0.72
Rodentia	family	18/32	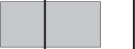	0.66	−0.95
*Rodentia*	*genus*	*82/450*	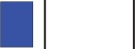	*−1.81*	1.7*
*Rodentia*	*species*	*90/2094*	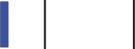	2.66**	2.36**
Scandentia	family	2/2	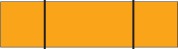		
Scandentia	genus	2/5	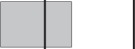	−0.77	−0.76
Scandentia	species	3/20	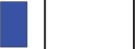	−2	−0.8
Sirenia	family	2/2	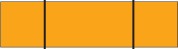		
Sirenia	genus	2/2	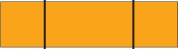		
Sirenia	species	4/4	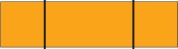		
Soricomorpha	family	3/4	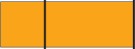	−0.98	−0.97
*Soricomorpha*	*genus*	*19/43*	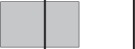	7.07**	2.64**
*Soricomorpha*	*species*	*21/392*	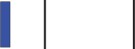	10.17**	3.36**
Tubulidentata	family	1/1	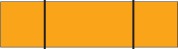		
Tubulidentata	genus	1/1	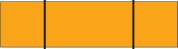		
Tubulidentata	species	1/1	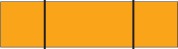		

Across mammals, taxa with available coded anatomical characters were significantly clustered using NTI at the species- and genus-level. For each order, only seven showed significant clustering (Cetartiodactyla, Cingulata, Pilosa and Rodentia at the species-level, and Carnivora, Chiroptera and Soricomorpha at both species- and genus-level) and none showed significant overdispersion (table 1).

**Figure 1.**
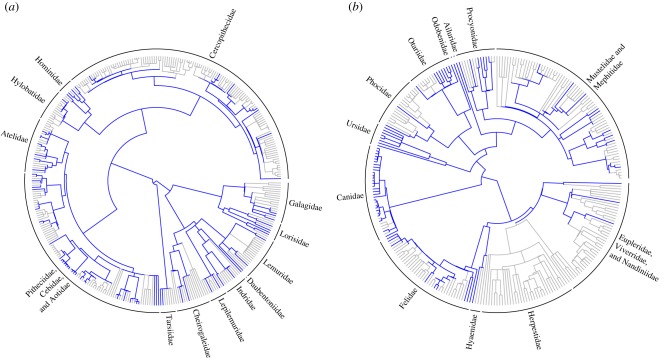
Phylogenetic distribution of species with available coded anatomical characters across two orders (*a*, Primates; *b*, Carnivora). Blue branches indicate species with available coded anatomical characters.

Figure 1 shows randomly distributed OTUs with available coded anatomical characters in Primates (figure 1*a*) and phylogenetically clustered OTUs with available coded anatomical characters in Carnivora (mainly Canidae and Ursidae but no Herpestidae; figure 1*b*).

## 5. Discussion

Our results show that although phylogenetic relationships among living mammals are well resolved [10,16], most of the data used to build these phylogenies are molecular, and few coded anatomical characters are available for living mammals compared with fossils [17,18]. This has implications for building total evidence phylogenies, as without sufficient overlapping anatomical characters for living and fossil species, fossil placements in these trees may be unreliable [8].

The number of living mammalian OTUs with available coded anatomical characters was surprisingly low at the species-level: only 17%. Only seven out of 28 orders have a high coverage of taxa with available coded anatomical characters. This high coverage threshold of 75% of taxa with available characters represents the minimum amount of data required before missing data have a significant effect on the topology of total evidence trees [8]. Beyond this threshold, there is considerable displacement of wildcard taxa and decreased clade conservation [8]. Therefore, we expect difficulties in placing fossils at the species-level in most mammalian orders, but fewer issues at higher taxonomic levels. Additionally, our analyses may underestimate the problem as we do not distinguish between soft and hard tissue characters; if a living taxon has only soft tissue coded anatomical characters, then it will not have overlapping data with fossils that only have hard tissues preserved.

When few species have available coded anatomical characters, the ideal scenario is for them to be evenly distributed (as measured by phylogenetic overdispersion) to maximize the possibilities of a fossil being placed in the correct clade. The second best scenario is that species with available characters are randomly distributed across the phylogeny. Here, we expect no bias in the placement of fossils [8], and it is therefore encouraging that for most orders, species with available coded anatomical characters were randomly distributed across the phylogeny. The worst-case scenario for fossil placement is that species with available characters are phylogenetically clustered. Then, we expect two major biases: first, fossils will not be placed within a clade containing no hard tissue data, and second, fossils will have higher probability of being placed within the most sampled clade by chance. Our results suggest that this may be problematic at the genus-level in Carnivora, Chiroptera and Soricomorpha. For example, a carnivoran fossil is unlikely to be placed in herpestidae because they have no coded anatomical characters available. Instead, the fossil will have a high probability of being placed on a branch that contains many anatomical characters, such as within the Canidae or Ursidae (figure 1*b*). This is analogous to the problem of long-branch attraction/short-branch repulsion, as one can think of Herpestidae as having zero-length branches for anatomical characters, and Canidae and Ursidae having long branches and thus ‘attracting’ fossil placements.

We acknowledge, however, that our analysis does not include all matrices containing anatomical characters ever published. Instead, our data collection procedure focused on including studies that provided easily accessible matrices, i.e. we did not include matrices that are only available in books, non-reusable formats (e.g. an image of the matrix) or matrices available only upon request from the authors. Matrices containing anatomical characters were more common before the advent of molecular phylogenetics, but these matrices are also more likely to be unavailable in a reusable format, thus will be missing from our analyses. Although this will bias our results towards lower coverage we do not think this bias will be large, as many recent morphological matrices reuse living taxa characters from older matrices (see electronic supplementary material, S1), so many of these data will be present in our analyses. Additionally, these older matrices are likely to differ from more recent ones in terms of their underlying definition of homology and their coding practices (see [19]). Therefore, care needs to be taken when deciding how to include these older matrices.

Despite the absence of good morphological/anatomical data coverage for living mammals, the total evidence method still seems to be the most promising way of combining living and fossil species in macroevolutionary analyses. Following the recommendations in [8], we should code anatomical characters for as many living species as possible. Fortunately, mammal specimens are usually readily available in natural history collections, therefore, we propose increased effort into coding anatomical characters from living species, possibly by engaging in collaborative data collection projects. Such efforts would be valuable not only to phylogeneticists, but also to any researcher focusing on understanding macroevolutionary patterns and processes.

## Supplementary Material

ESM 1

## Supplementary Material

ESM 2

## Data accessibility

The original data for this analysis are available on Dryad (provisional doi:10.5061/dryad.qj552). All data and code for repeating the analysis are available on GitHub (https://github.com/TGuillerme/Missing_living_mammals).
