# Supplementary material for: Assessment of available anatomical characters for linking living mammals to fossil taxa in phylogenetic analyses
Source: Biol Lett. 2016 May;12(5):20151003. doi: 10.1098/rsbl.2015.1003 (PMC4892235; doi:10.1098/rsbl.2015.1003)
Supplement: ESM 1 [file rsbl20151003supp1.pdf]

Version dated: March 29, 2016

Assessment of available anatomical characters for linking living mammals to fossil taxa in phylogenetic analyses

Electronic Supplementary Material 1

Thomas Guillerme[1,*] and Natalie Cooper[1,2]

[1]*School of Natural Sciences, Trinity College Dublin, Dublin 2, Ireland.*

[2]*Department of Life Sciences, Natural History Museum, Cromwell Road, London, SW7 5BD, UK.*

***Corresponding author.** *t.guillerme@imperial.ac.uk*

1 - Data collection

Public repositories

We downloaded available matrices containing fossil and/or living mammal taxa from the three databases using the following list of keywords:

`Mammalia; Monotremata; Marsupialia; Placentalia; Macroscelidea; Afrosoricida; Tubulidentata; Hyracoidea; Proboscidea; Sirenia; Pilosa; Cingulata; Scandentia; Dermoptera; Primates; Lagomorpha; Rodentia; Erinaceomorpha; Soricomorpha; Cetacea; Artiodactyla; Cetartiodactyla; Chiroptera; Perissodactyla; Pholidota; Carnivora; Didelphimorphia; Paucituberculata; Microbiotheria; Dasyuromorphia; Peramelemorphia; Notoryctemorphia; Diprotodontia`.

Details about the specific search options used for each public repository are listed below. Note that some matrices were downloaded from more than one database but this is not a problem because we are interested in the total number of unique living operational taxonomic units (OTUs), therefore even if some were present in more than one matrix they still only counted as a single OTU.

MorphoBank.— We accessed the MorphoBank repository (`morphobank.org`) on 10th June 2015 and used the keywords listed above in the search menu. We downloaded the data associated with each project matching with the keyword.

Graeme Lloyd.— We accessed Graeme Lloyd's website repository (`graemetlloyd.com/`) on 10th June 2015 and downloaded all the matrices that were available with a direct download link in the mammal data section of the website (`graemetlloyd.com/matrmamm.html`).

Ross Mounce.— We accessed Ross Mounce's GitHub repository (`github.com/rossmounce/cladistic-data`) on 11th June 2015 and downloaded all 601 matrices. We then ran a shell script to select only the matrices that had any text element that matched with one of the search terms (`github.com/TGuillerme/Missing_living_mammals/blob/master/Functions/select.files.sh`). To make the matrix selection more thorough, we ignored the case and Latin suffix (i.e. *ia*, *ata*, *ea*, and *a*) of the keywords.

Google Scholar (accessed 11th June 2015)

To ensure we did not miss any extra matrices that were not available on one of these repositories, we ran a Google Scholar search on the 11th June using the following keywords:

`order ("morphology" OR "morphological" OR "cladistic") AND characters matrix paleontology phylogeny`

where *order* was replaced by each of the taxonomic subdivision keywords listed above in turn. For each taxonomic subdivision keyword we selected the 20 first papers published since 2010 resulting in 660 papers. We selected only the 20 first results for

each search term to avoid downloading large numbers of irrelevant articles, and because the rate of discovery of new matrices was very low and unlikely to be substantially improved by downloading more papers. For example, in the 660 papers we downloaded, only 50 contained extra living OTUs and only contributed 425 OTUs to our total of 4950 OTUs (Figure 1). We selected only articles published since 2010 because almost every recently published matrices contained some of the morphological characters and OTUs from previous studies, thus almost all older studies are represented in the matrices we collected. For example, the six living primates used in [1] (*Aotus trivirgatus, Galago demidoff, Lemur catta, Microcebus murinus, Nycticebus coucang and Saimiri sciureus*) and their associated characters are reused along with more living species and characters in [2, 3, 4, 5, 5, 6, 7, 8, 9, 10, 11, 12, 13].

The list of all 286 downloaded matrices is available on `github.com/TGuillerme/Missing_living_mammals/tree/master/Data/Matrices`. The matrices contained a total of 11010 operational taxonomic units (OTUs) of which 5228 were unique. In this study, we refer to OTUs rather than species because the entries in the downloaded matrices were not standardised and ranged from specific individual specimen names (i.e. the name of a collection item) to the family-level. Where possible, we considered OTUs at their lowest valid taxonomic level (i.e. species) but some OTUs were only valid at a higher taxonomic level (e.g. genus or family). Therefore for some orders, we sampled more genera than species.

Standardising the matrices

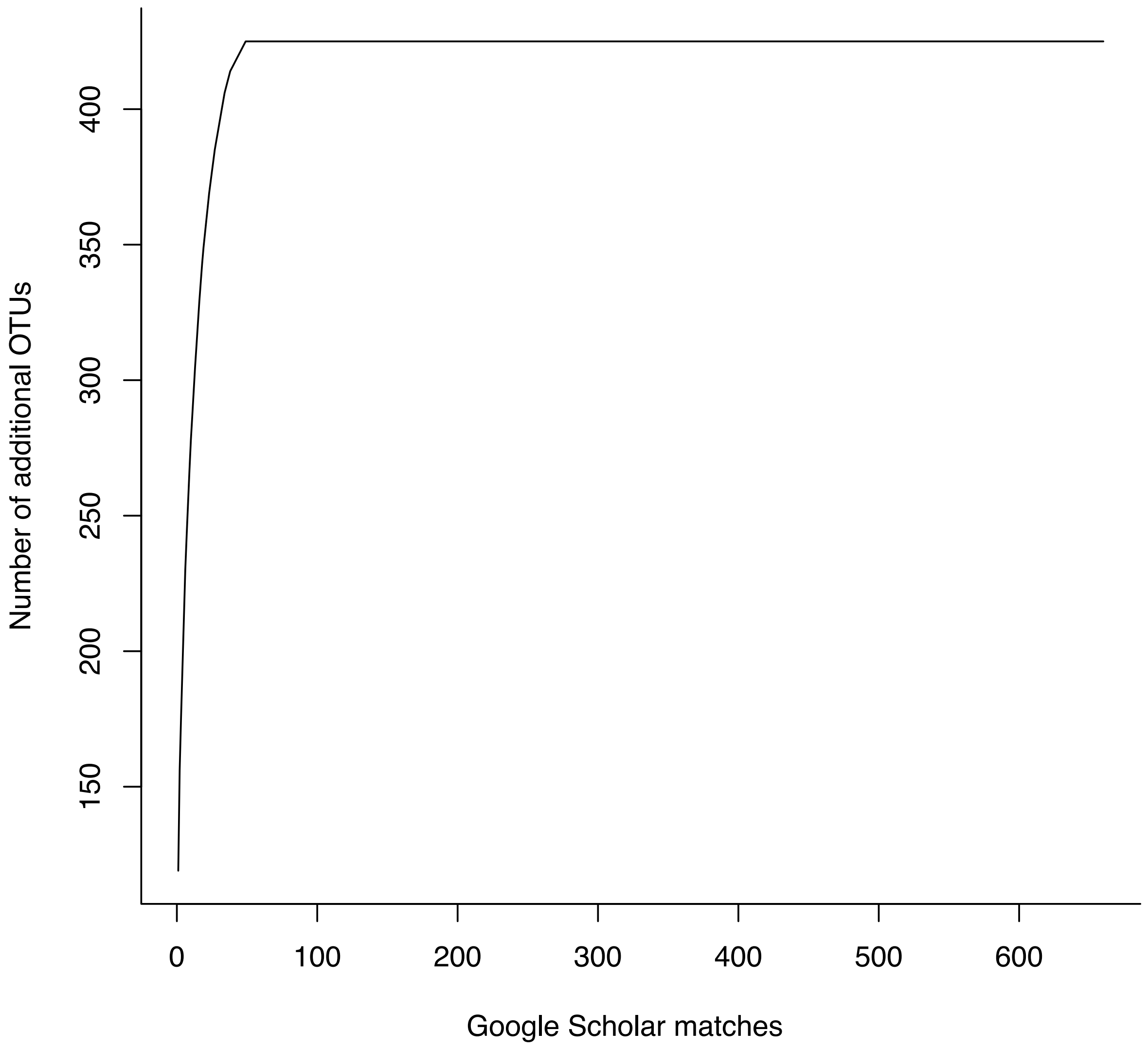

Figure 1: Google Scholar searches additional OTUs rarefaction curve. The x-axis represents the number of Google Scholar matches (papers, books or abstracts) and the y-axis represents the cumulative number of additional living OTUs for each Google Scholar match.

We transformed all the non-NEXUS matrices (TNT, Word, Excel, JPEG) to NEXUS format manually. We then cleaned the NEXUS matrices by removing any extra information (trees, continuous characters, morphological character descriptions, molecular data) to end up with NEXUS matrices containing only the discrete morphological data. We then manually fixed the incorrectly-formatted binomial names (e.g. *H. sapiens* became *Homo sapiens*) using the abbreviation list in the relevant publications. All the standardised matrices are available on `github.com/TGuillerme/Missing_living_mammals/tree/master/Data/Matrices_binomial/Matrices`.

Selecting the living OTUs

We designated as "living" all OTUs that were either present in the phylogeny of [14] or the taxonomy of [15], and designated as "fossil" all OTUs that were present in the Paleobiology database (`paleobiodb.org/`). For OTUs that did not appear in these three sources, we first decomposed the name (i.e. *Homo sapiens* became *Homo* and *sapiens*) and tried to match the first element with a higher taxonomic level (family, genus etc.). Any OTUs that still had no matches in the sources above were designated as non-applicable (NA; Figure 2). Non-applicable OTUs were either specimen IDs with no related taxon names (e.g. *FMNHPR2081*), abbreviations that were not described in the associated paper (e.g. *Ho.sap.*), non-mammals *stricto-sensu* (e.g. *Sinoconodon*), non standard taxonomic levels (e.g. *Spalcotheriids*) invalid taxonomic designations (e.g. *sp_nov_1* or *Outgroup*) or typos (e.g. *Hobo sapions*).

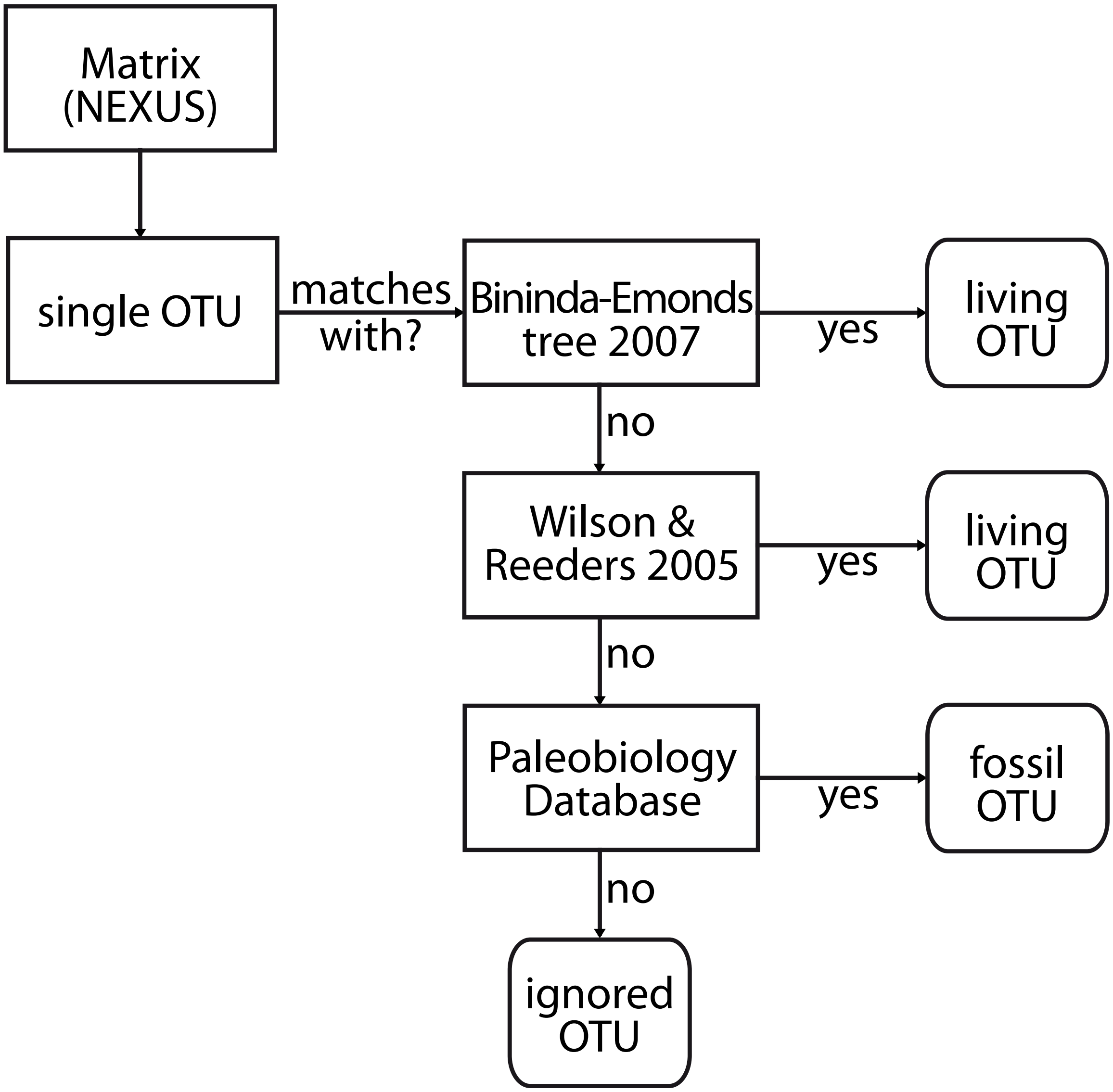

Figure 2: Taxonomic matching algorithm used in this study. For each matrix, each operational taxonomic unit (OTU) is matched with the supertree from Fritz et al. 2009. If the OTU matches, then it is classified as living. Otherwise it is matched with the Wilson & Reeder 2005 mammalian taxonomy. If the OTU matches, then it is classified as living. Otherwise it is matched with the Paleobiology database list of mammals. If the OTU matches, then it is classified as fossil. Otherwise it is ignored.

2 - Data collection reproducibility

Every step of the analysis (apart from downloading and standardisation of the matrices) is entirely repeatable via GitHub (`github.com/TGuillerme/Missing_living_mammals`).

*

References

[1] Ross C, Williams B, Kay RF. Phylogenetic analysis of anthropoid relationships. J Hum Evol. 1998;35(3):221–306.

[2] Seiffert ER, Simons EL, Attia Y. Fossil evidence for an ancient divergence of lorises and galagos. Nature. 2003;422(6930):421–424.

[3] Marivaux L, Antoine PO, Baqri SRH, Benammi M, Chaimanee Y, Crochet JY, et al. Anthropoid primates from the Oligocene of Pakistan (Bugti Hills): data on early anthropoid evolution and biogeography. Proc Nat Acad Sci. 2005;102(24):8436–8441.

[4] Seiffert ER, Simons EL, Clyde WC, Rossie JB, Attia Y, Bown TM, et al. Basal anthropoids from Egypt and the antiquity of Africa's higher primate radiation. Science. 2005;310(5746):300–304.

[5] Bloch JI, Silcox MT, Boyer DM, Sargis EJ. New Paleocene skeletons and the relationship of plesiadapiforms to crown-clade primates. Proc Nat Acad Sci. 2007;104(4):1159–1164.

[6] Kay RF, Fleagle J, Mitchell T, Colbert M, Bown T, Powers DW. The anatomy of Dolichocebus gaimanensis, a stem platyrrhine monkey from Argentina. J Hum Evol. 2008;54(3):323–382.

[7] Silcox MT. The biogeographic origins of Primates and Euprimates: east, west, north, or south of Eden? In: Mammalian Evolutionary Morphology. Springer; 2008. p. 199–231.

[8] Seiffert ER, Perry JM, Simons EL, Boyer DM. Convergent evolution of anthropoid-like adaptations in Eocene adapiform primates. Nature. 2009;461(7267):1118–1121.

[9] Tabuce R, Marivaux L, Lebrun R, Adaci M, Bensalah M, Fabre PH, et al. Anthropoid versus strepsirhine status of the African Eocene primates Algeripithecus and Azibius: craniodental evidence. P Roy Soc B-Biol Scis. 2009;p. rspb20091339.

[10] Boyer DM, Seiffert ER, Simons EL. Astragalar morphology of Afradapis, a large adapiform primate from the earliest late Eocene of Egypt. Am J Phys Anthropol. 2010;143(3):383–402.

[11] Seiffert ER, Simons EL, Boyer DM, Perry JM, Ryan TM, Sallam HM. A fossil primate of uncertain affinities from the earliest late Eocene of Egypt. Proc Nat Acad Sci. 2010;107(21):9712–9717.

[12] Marivaux L, Ramdarshan A, Essid EM, Marzougui W, Ammar HK, Lebrun R, et al. Djebelemur, a tiny pre-tooth-combed primate from the Eocene of Tunisia: a glimpse into the origin of crown strepsirhines. PloS ONE. 2013;8(12):e80778.

[13] Ni X, Gebo DL, Dagosto M, Meng J, Tafforeau P, Flynn JJ, et al. The oldest known primate skeleton and early haplorhine evolution. Nature. 2013;498(7452):60–64.

[14] Bininda-Emonds OR, Cardillo M, Jones KE, MacPhee RD, Beck RM, Grenyer R, et al. The delayed rise of present-day mammals. Nature. 2007;446(7135):507–512. Available from: `http://dx.doi.org/10.1038/nature05634`.

[15] Wilson DE, Reeder DM. Mammal species of the world: a taxonomic and geographic reference. vol. 1. JHU Press; 2005.
