# Supplementary material for: Assessment of available anatomical characters for linking living mammals to fossil taxa in phylogenetic analyses
Source: Biol Lett. 2016 May;12(5):20151003. doi: 10.1098/rsbl.2015.1003 (PMC4892235; doi:10.1098/rsbl.2015.1003)
Supplement: ESM 2 [file rsbl20151003supp2.pdf]

Version dated: March 29, 2016

Assessment of available anatomical characters for linking living mammals to fossil taxa in phylogenetic analyses

Electronic Supplementary Material 2

Thomas Guillerme[1,*] and Natalie Cooper[1,2]

[1]*School of Natural Sciences, Trinity College Dublin, Dublin 2, Ireland.*

[2]*Department of Life Sciences, Natural History Museum, Cromwell Road, London, SW7 5BD, UK.*

***Corresponding author.** *t.guillerme@imperial.ac.uk*

Supplementary results - 1

The following section contains a supplementary analysis looking at the sampling effort for mammalian orders at three different taxonomic levels (family, genus and species). We fitted linear regressions between the log of the number of sampled families/genera/species for each order against the log of the total number of families/genera/species in each order from [1] and fixed the intercept at zero (because when no families/genera/species exist they cannot be sampled). The slope of the regression gives an indication of the sampling effort: if the slope is 1, the number of sampled families/genera/species and the total number of families/genera/species are equal, i.e. all families/genera/species have been sampled. Slopes of less than 1 indicate lower sampling effort. The code for this analysis is available here: `github.com/TGuillerme/Missing_living_mammals/blob/master/Analysis/Sampling_effort.R`.

This analysis suggests that sampling effort effectively reflects diversity for each taxonomic level, i.e. morphological characters were collected for each order according to its size (Figure 1). However, it is worth noting that this relation is stronger at higher taxonomic levels (i.e. family-level, adjusted R^2=0.99 b=0.936) compared to the species-level (R^2=0.846 b=0.71). These results might be simply due to the increasing number of groups at each taxonomic level (i.e. number of species $>$ genera $>$ families). But it is also probable that mammalian morphological phylogeneticists have focused on resolving higher level relationships among mammalian orders (see Table 1 in the main text), resulting in better coverage at the family-level.

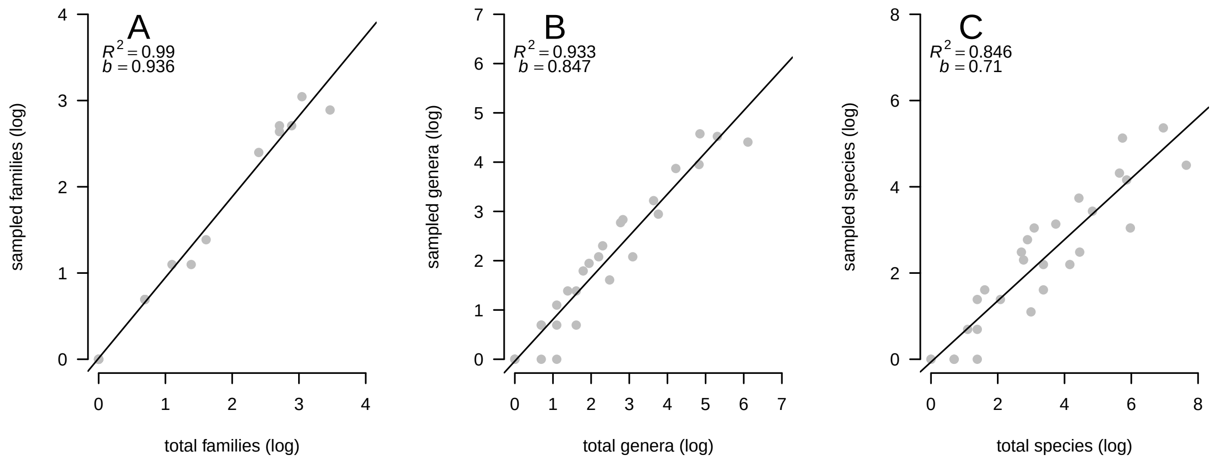

Figure 1: Sampling effort across mammalian orders at the family (A), genus (B) and species (C) levels. The R^2 (adjusted) and the slope (*b*, forced through the origin) are reported for each regression.

*

References

[1] Bininda-Emonds OR, Cardillo M, Jones KE, MacPhee RD, Beck RM, Grenyer R, et al. The delayed rise of present-day mammals. Nature. 2007;446(7135):507–512. Available from: `http://dx.doi.org/10.1038/nature05634`.

Supplementary results - 2

The following section contains phylogenetic representations of availability of coded anatomical characters for each mammalian order (excluding results for Primates and Carnivora that are present in the main text).

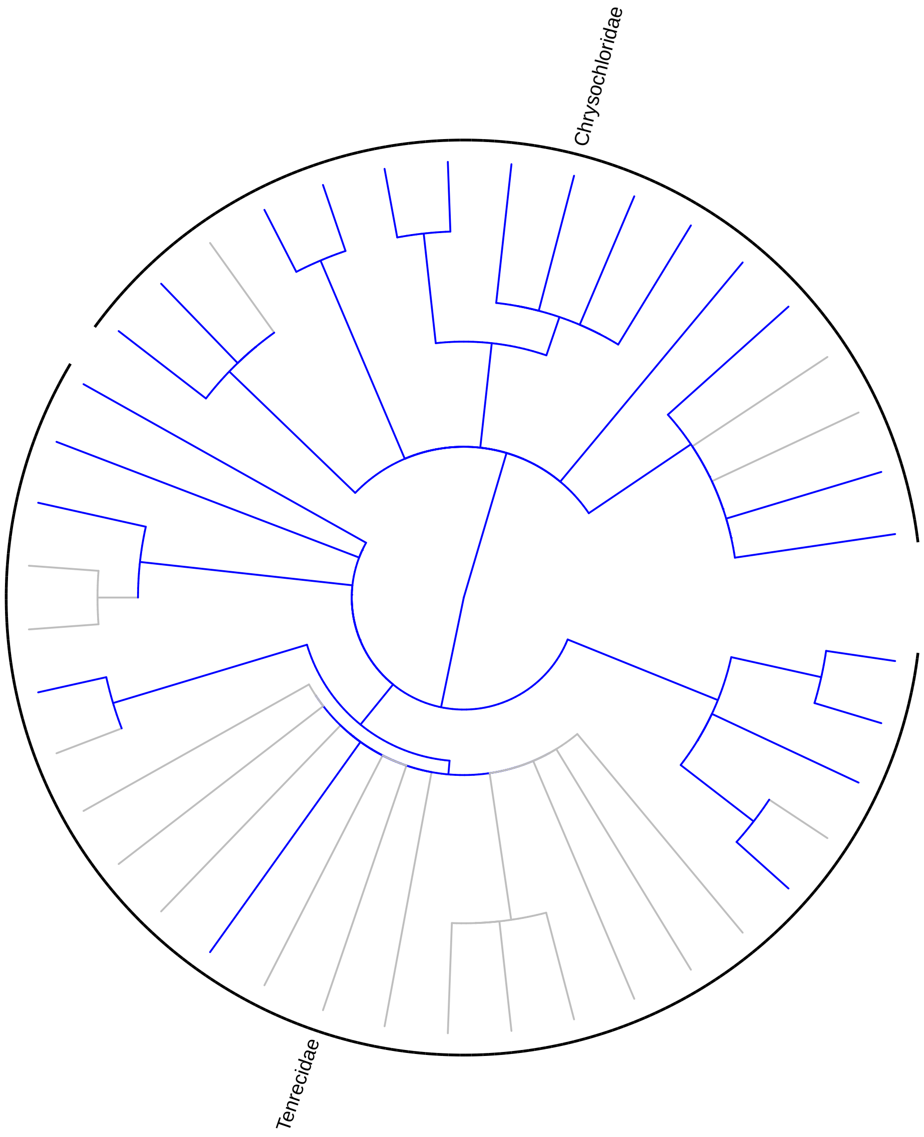

Figure 2: Phylogenetic distribution of species with available coded anatomical characters across Afrosoricida. Blue branches indicate species with available coded anatomical characters.

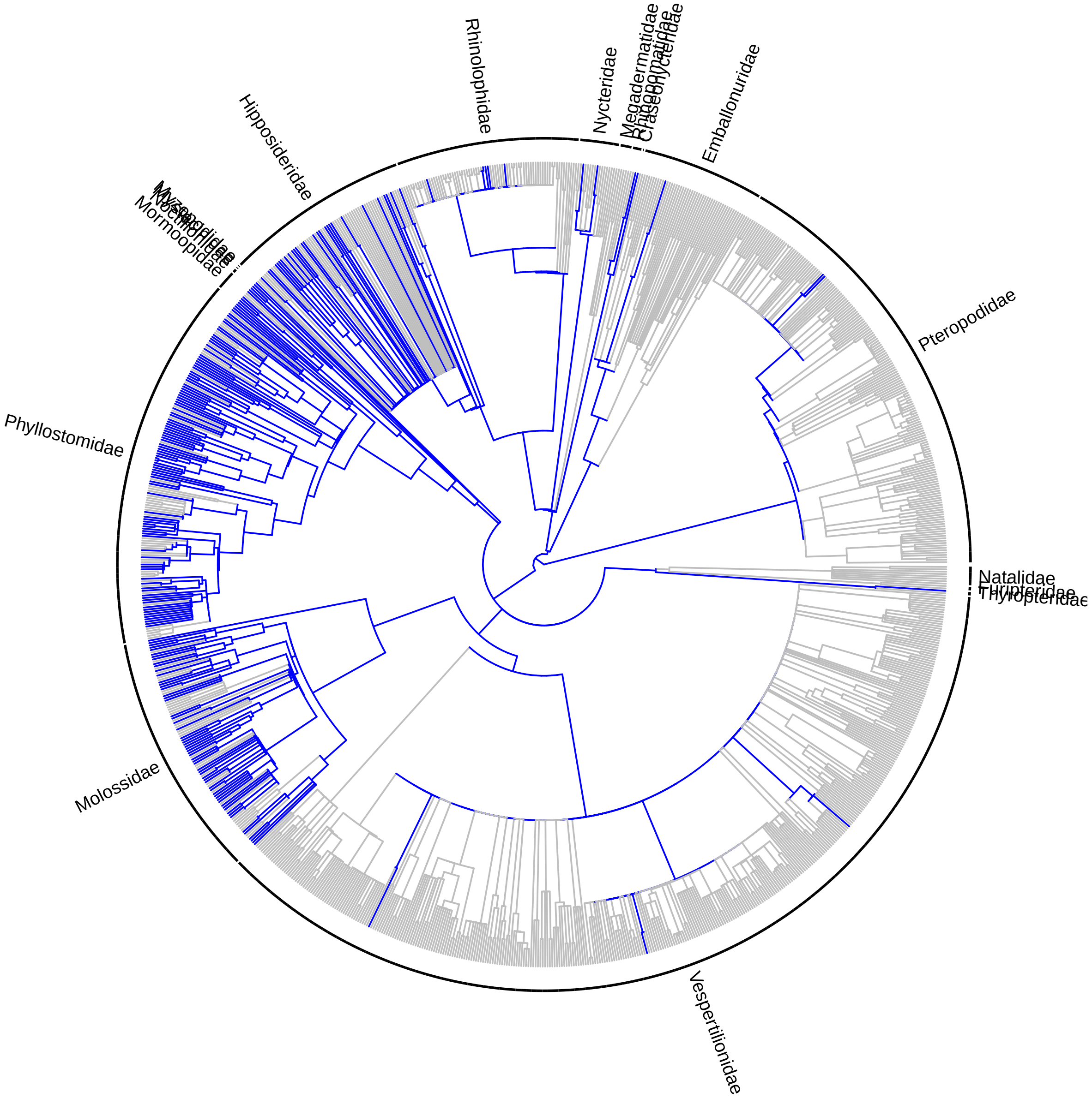

Figure 3: Phylogenetic distribution of species with available coded anatomical characters Chiroptera. Blue branches indicate species with available coded anatomical characters

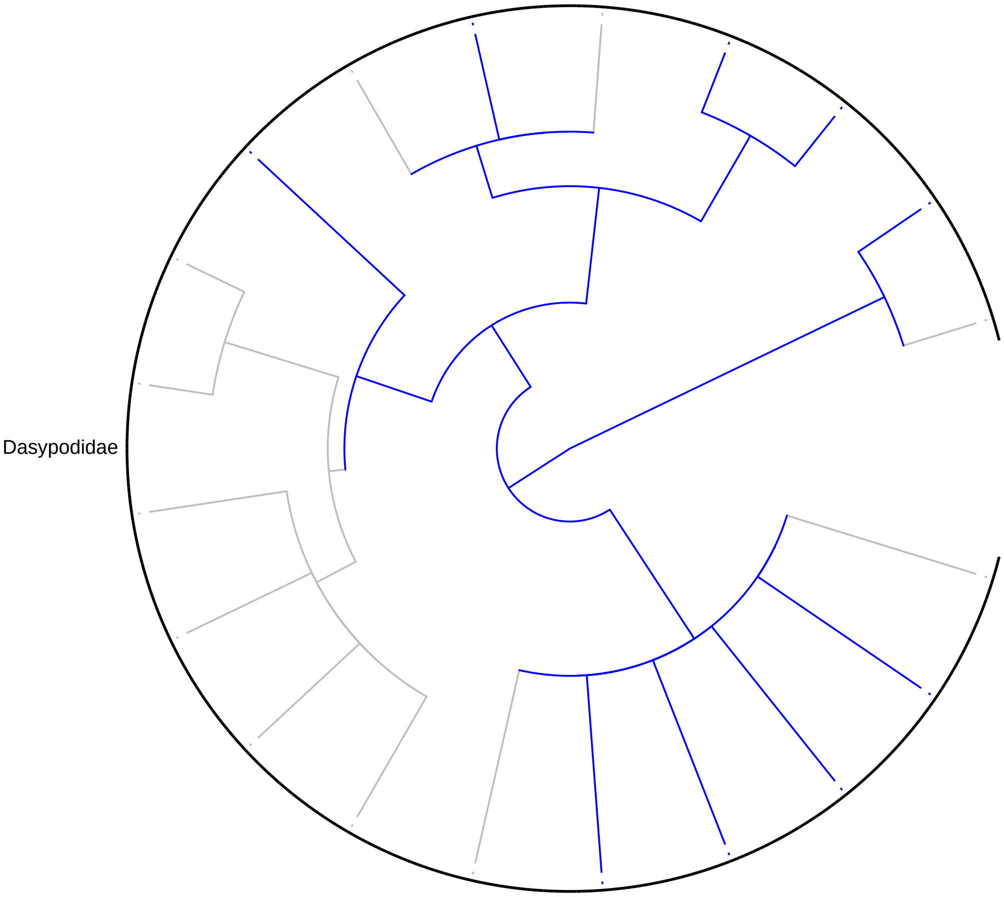

Figure 4: Phylogenetic distribution of species with available coded anatomical characters

Cingulata. Blue branches indicate species with available coded anatomical characters

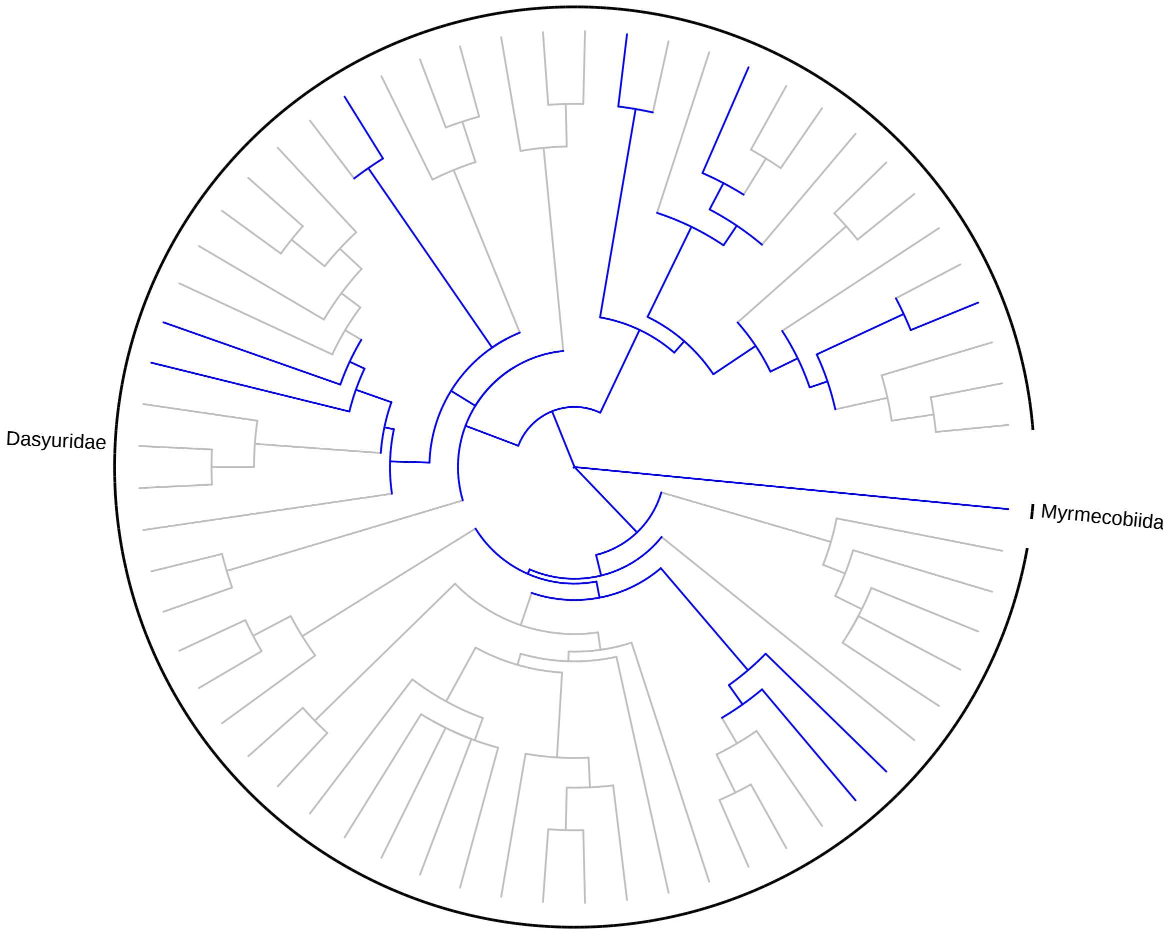

Figure 5: Phylogenetic distribution of species with available coded anatomical characters Dasyuromorphia. Blue branches indicate species with available coded anatomical characters

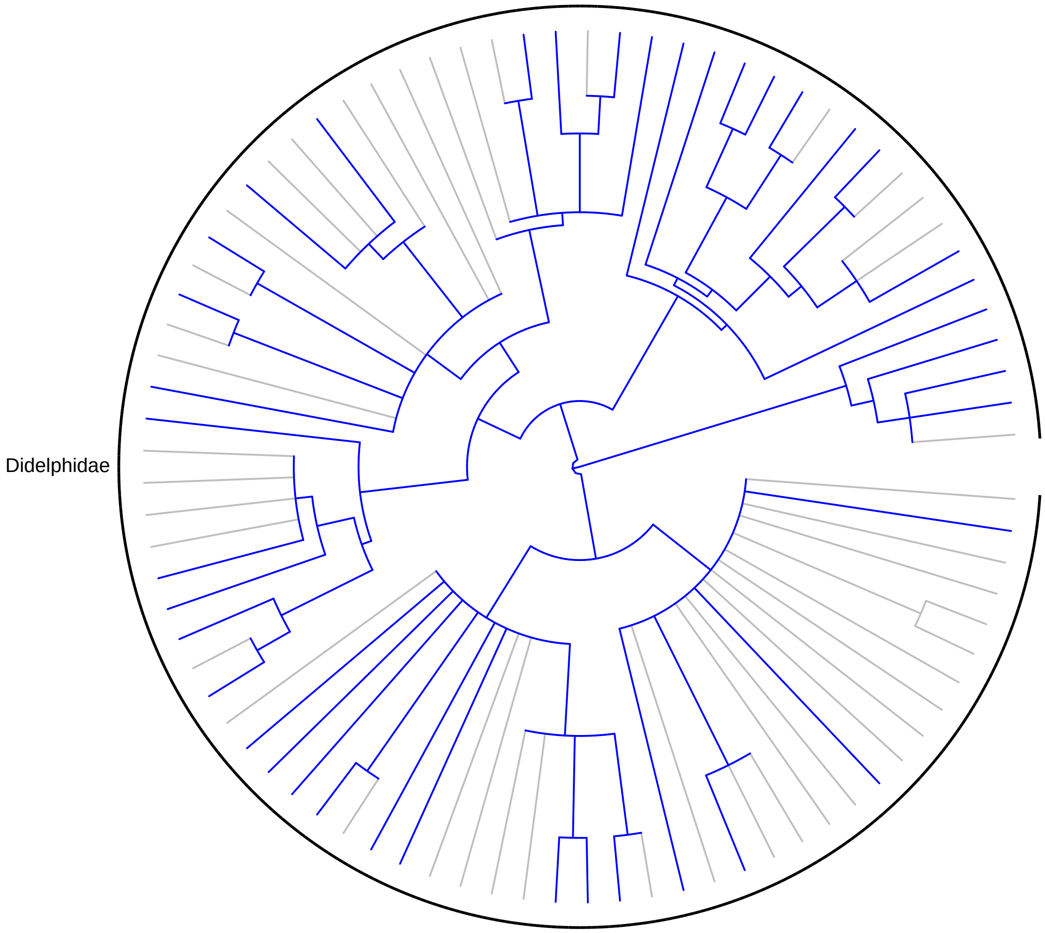

Figure 6: Phylogenetic distribution of species with available coded anatomical characters Didelphimorphia. Blue branches indicate species with available coded anatomical characters

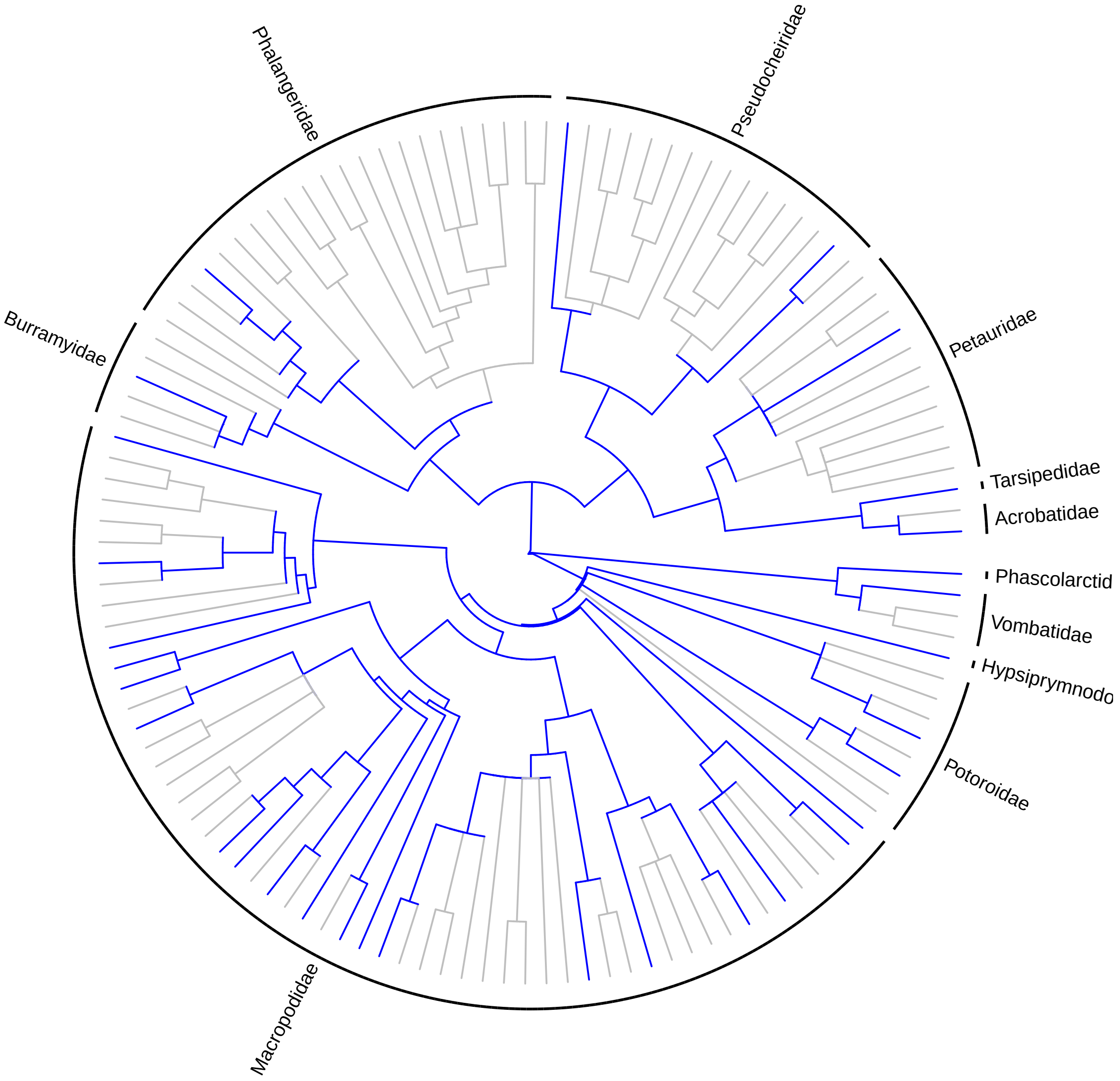

Figure 7: Phylogenetic distribution of species with available coded anatomical characters Diprotodontia. Blue branches indicate species with available coded anatomical characters

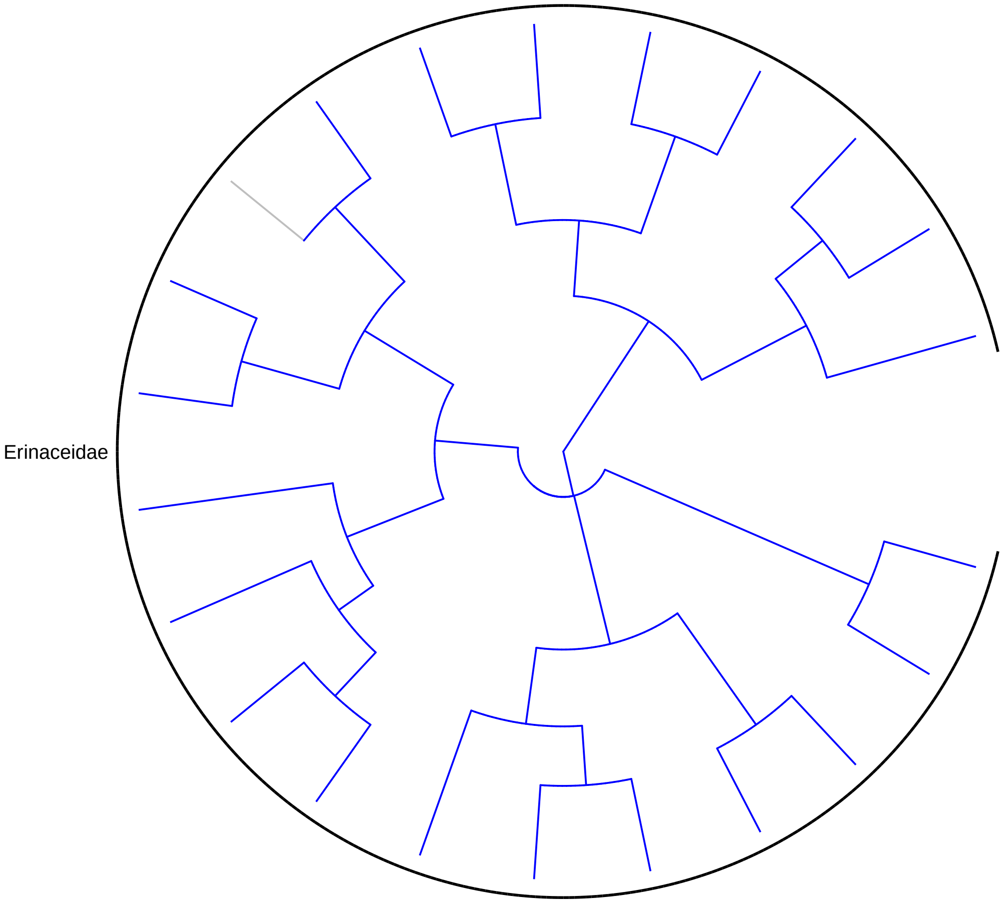

Figure 8: Phylogenetic distribution of species with available coded anatomical characters Erinaceomorpha. Blue branches indicate species with available coded anatomical characters

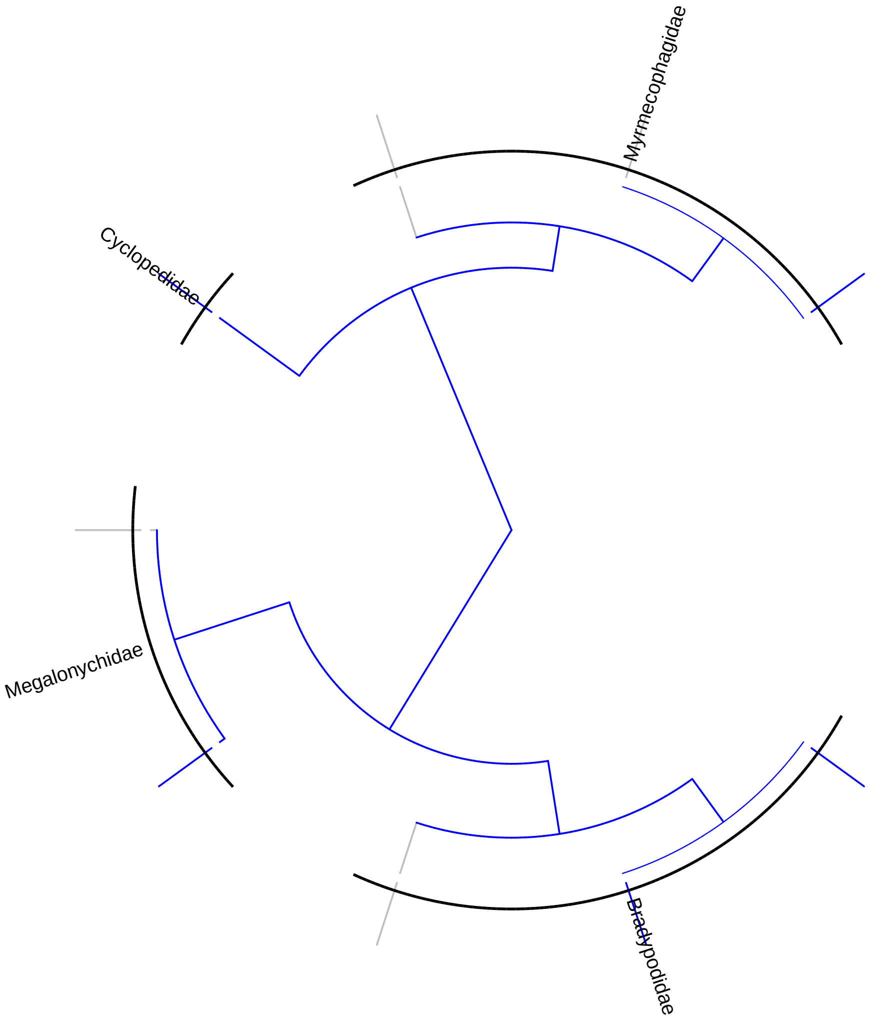

Figure 9: Phylogenetic distribution of species with available coded anatomical characters

Pilosa. Blue branches indicate species with available coded anatomical characters

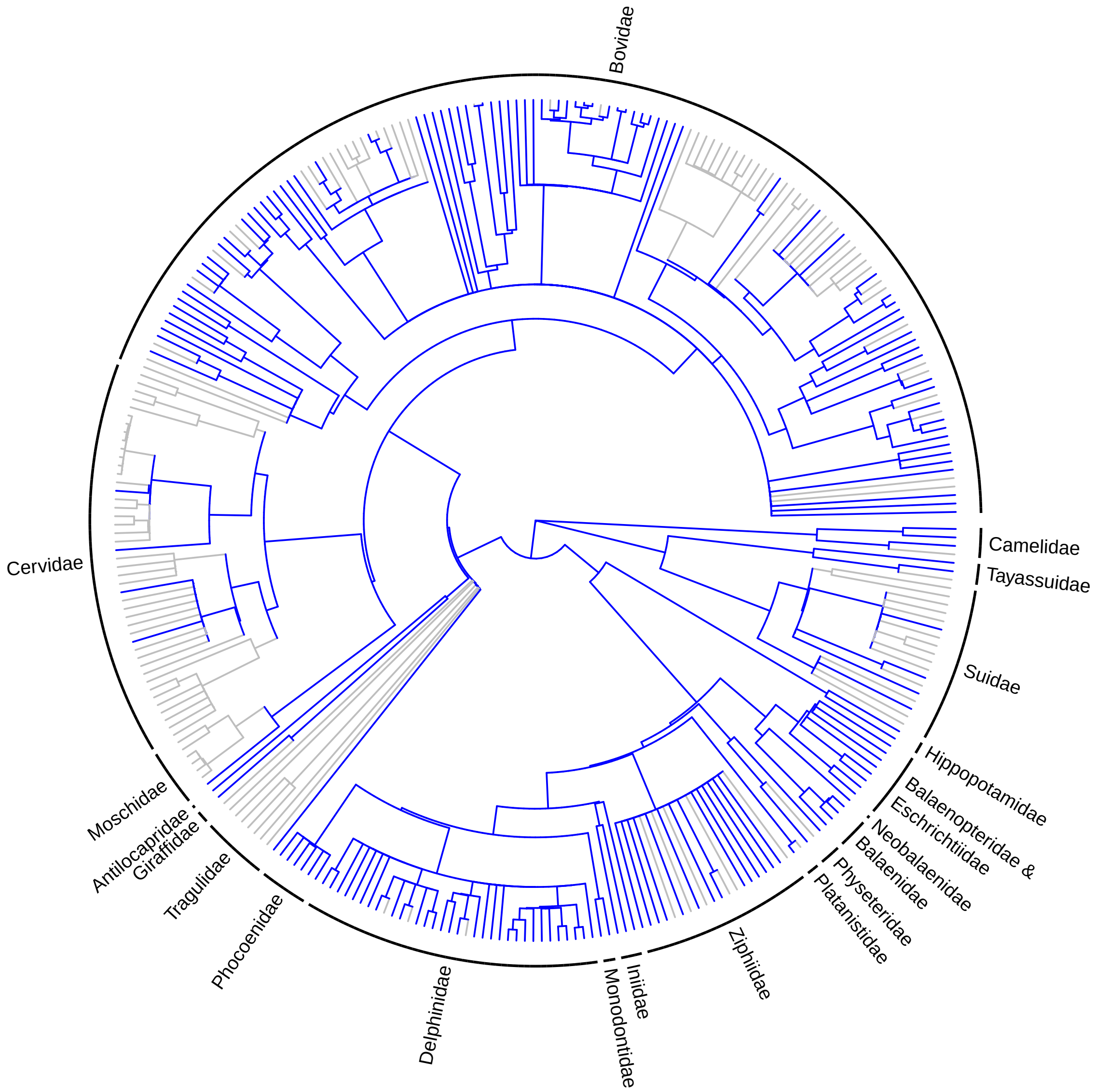

Figure 10: Phylogenetic distribution of species with available coded anatomical characters Cetartiodactyla. Blue branches indicate species with available coded anatomical characters

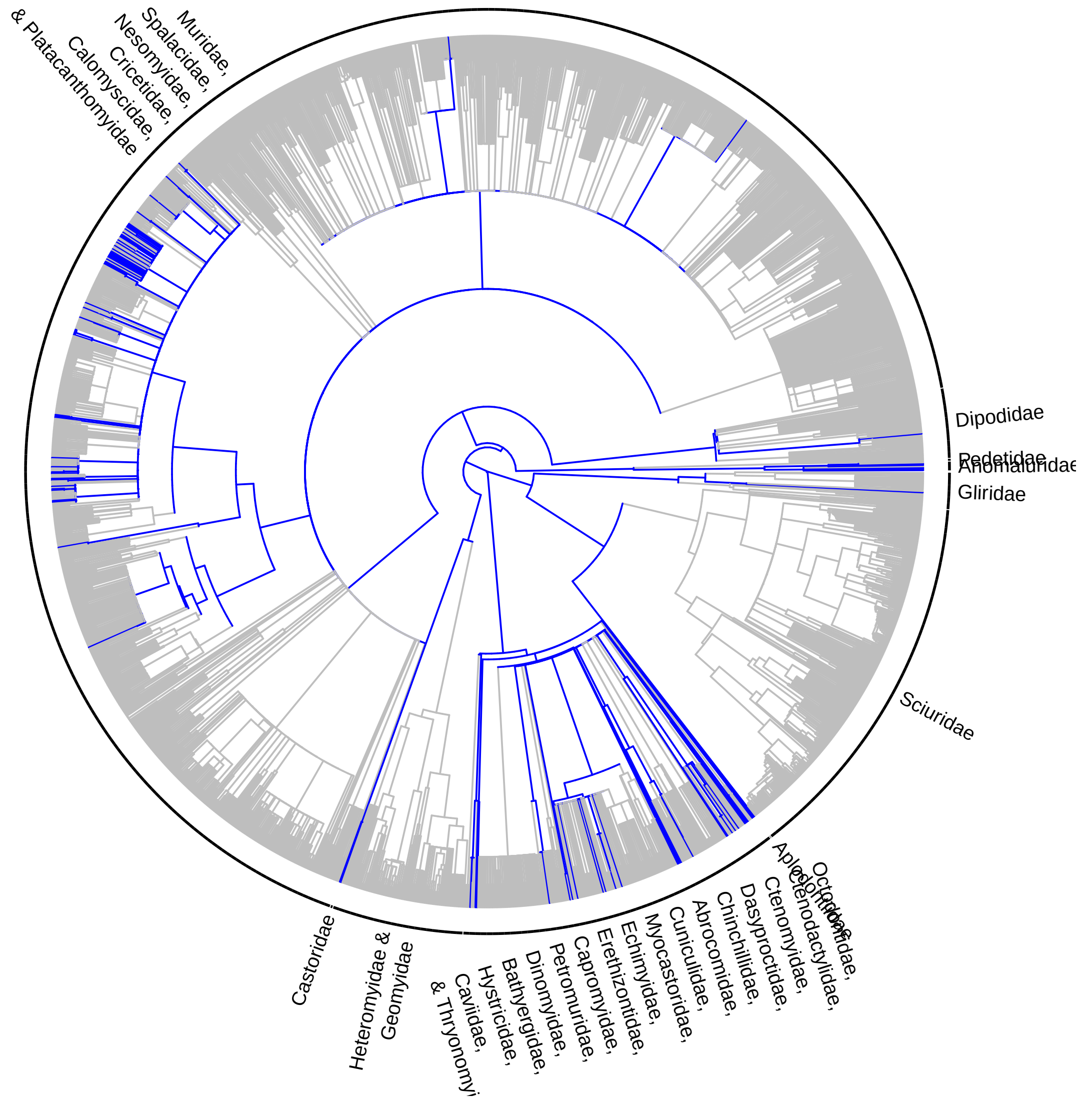

Figure 11: Phylogenetic distribution of species with available coded anatomical characters Rodentia. Blue branches indicate species with available coded anatomical characters

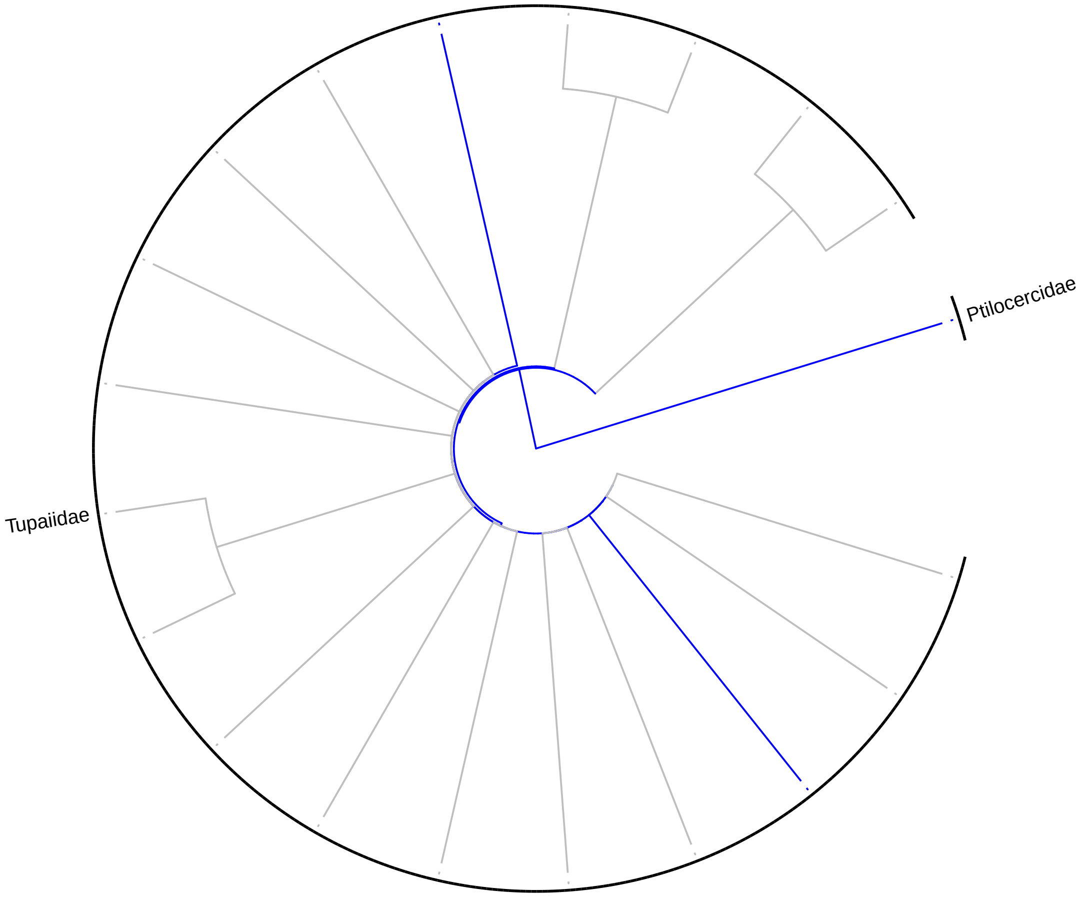

Figure 12: Phylogenetic distribution of species with available coded anatomical characters Scandentia. Blue branches indicate species with available coded anatomical characters

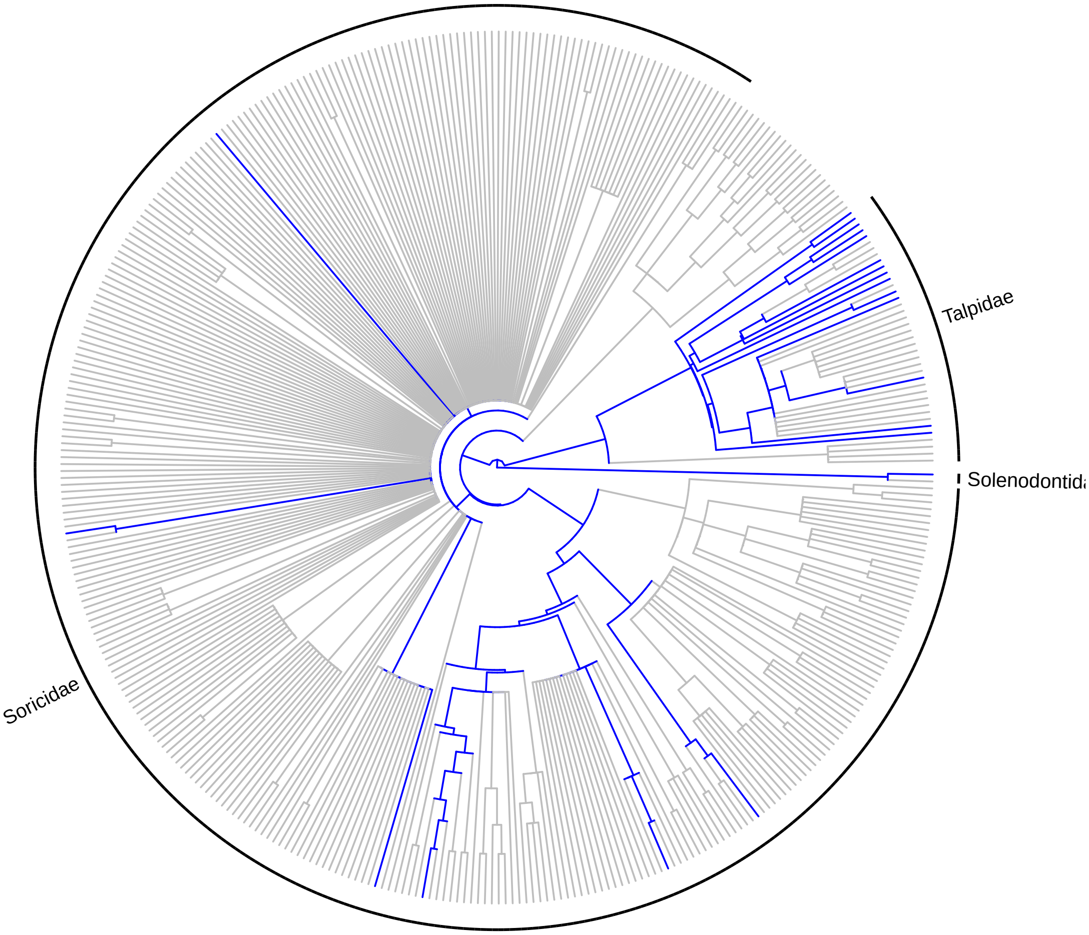

Figure 13: Phylogenetic distribution of species with available coded anatomical characters Soricomorpha. Blue branches indicate species with available coded anatomical characters
